# Electronic medication monitor for people with tuberculosis: Implementation experience from thirty counties in China

**DOI:** 10.1371/journal.pone.0232337

**Published:** 2020-04-29

**Authors:** Ni Wang, Hemant Deepak Shewade, Pruthu Thekkur, Fei Huang, Yanli Yuan, Xiaomeng Wang, Xiaolin Wang, Miaomiao Sun, Hui Zhang

**Affiliations:** 1 National Center for Tuberculosis Control and Prevention, Chinese Center for Disease Control and Prevention, Beijing, China; 2 International Union against Tuberculosis and Lung Disease (The Union), Paris, France; 3 The Union South East Asia Office, New Delhi, India; 4 Jilin Research Institute of Tuberculosis Control, Changchun, China; 5 Zhejiang Provincial Center for Disease Control and Prevention, Hangzhou, China; 6 The Fourth People’s Hospital of Ningxia Hui Autonomous Region, Yinchuan, China; 7 PATH China Program, Beijing, China; Imperial College London, UNITED KINGDOM

## Abstract

**Background:**

China piloted a digital adherence technology called electronic medication monitor (EMM) to support self-administered treatment for tuberculosis. EMM is a portable plastic box that records each time the device is opened, offering an indirect measure of treatment adherence. During the monthly patient visits to tuberculosis designated hospitals, the doctors access the data from the EMM.

**Objectives:**

Among people with tuberculosis notified in 30 counties in China (July-December 2018) where EMM supported self-administered treatment was suggested to all those eligible (no communication impairment, ambulatory care), we assessed the i) proportion eligible for using EMM ii) uptake of EMM and factors associated and iii) treatment adherence, including the proportion shifted to DOT.

**Methods:**

This was an observational study using secondary programme data. Single instance of ≥50% or continued instance of 20–49% monthly missed doses was the eligibility criteria to shift to DOT. We used log binomial regression to identify factors associated with not using EMM within first month.

**Results:**

Of 2227 with EMM eligibility data, 1810 (81%) were eligible for EMM. Of 1810 people, 1314 (73%) ever used EMM anytime during treatment, among them, 134 (10%) were eligible for shift to DOT (based on EMM data), and 29 (22%) were shifted. In addition, 70 were shifted while the EMM data was missing. Of 1047 people who started using EMM within first month, we observed 6381 person-months of follow up and there were 1526(25%) instances of missing EMM data. Children (<15 years), elderly (≥65 years), semi-skilled or unemployed people, people with tuberculosis pleurisy and previous tuberculosis treatment were less likely to use EMM within first month.

**Conclusion:**

The EMM uptake was satisfactory but shift to DOT has to be ensured based on adherence data from EMM. The subsequent follow-up action when EMM data is missing has to be clarified in the guidelines.

## Introduction

Adherence to tuberculosis (TB) treatment may increase treatment success rates. In the last two decades, directly observed therapy (DOT) is the most widely used approach to ensure treatment adherence [1]. If DOT is not implemented systematically, it can result in poor patient adherence [2–4], which is a significant risk factor for unfavorable outcome [5]. Recent evidence shows practicality and feasibility issues in the implementation of DOT [1].

Patient-centered care with the selection of drug delivery platforms based on individual preferences (differential TB patient management) is being proposed to improve treatment adherence and success [1]. Self-administered treatment (SAT), along with adherence support systems, is one of the options. Digital adherence technologies like short message service (SMS, or text messaging), electronic medication monitor (EMM) and video observed treatment are the potential adherence support systems [6,7]. Compared with other digital technologies, EMMs are not dependent on mobile connectivity or internet coverage.

EMMs aim to provide patient flexibility by supporting them with instructions, dosing alerts and refill reminders. EMMs are automated electronic devices that record and inform the health care provider about the regularity with which a medicine container is opened (indirect measure of adherence) [6]. This provides objective information to the providers to make informed decisions on further adherence support, which includes shifting to DOT if required. Under trial conditions, EMM decreased the frequency of missed doses by 40% to 50% compared to usual treatment outside of the study as recommended by China’s national TB programme (NTP) [8]. Although the World Health Organization recommends EMM, the evidence is of very low certainty [6]. There is a need for generating evidence on how accurately an EMM’s records correlate with true treatment adherence [9], in addition to its acceptability, feasibility, effectiveness in increasing treatment success and role in improving health system efficiency before adoption and scale-up [10].

China has the world’s second-highest TB burden (nine percent of the global burden) [11]. Although the NTP recommends DOT for all people with TB, a systematic review found only 20% of people with TB received DOT from a health care worker, 30% received DOT from a family member, and 50% were on SAT without any adherence support [12]. Meanwhile, evidence showed that people on the SAT had poor adherence compared to those on DOT [4,8,13].

China’s 13th five-year plan on TB control (2016–2020) included the adoption of EMM as a part of differential patient management approaches. Previously, adherence support systems had been implemented for antiretroviral therapy in China under trial conditions[14], but none implemented under programmatic conditions. In China, there is a high level of acceptability and satisfaction with medication monitors among people with TB and health care workers [15,16]. Globally and in China, there is limited implementation experience under programmatic conditions regarding uptake of EMM, adherence to anti-TB treatment while using EMM and use of this information to improve treatment adherence under programmatic conditions.

We conducted this operational research among people with TB notified in 30 counties of China (July-December, 2018) where EMM supported SAT was suggested to all those eligible. We aimed to determine the i) proportion eligible to use EMM ii) proportion using EMM among those eligible and the factors associated with using EMM within the first month of diagnosis iii) and treatment adherence among those using EMM, including the proportion shifted to DOT.

## Methods

### Study design

We used a longitudinal descriptive design to assess the eligibility, uptake and treatment adherence and a cross-sectional analytical design to assess the factors associated with using EMM within the first month of diagnosis.

### Study setting

#### General setting

The mainland of China has a population of 1.4 billion, involving 31 provinces, 334 prefectures and 2851 counties. According to the geography and economy, the country is divided into eastern, middle and west regions. The economic development is higher in eastern regions and lower in western region. The prevalence of TB is higher in the western region compared to the eastern and middle regions. In 2017, there were an estimated 889 000 people with TB. The HIV prevalence among people with TB is low (2.1%) [11].

The national center for tuberculosis control and prevention, which belongs to the Chinese center for disease control and prevention (CDC), is in charge of NTP. TB management units are established at the provincial, prefecture and county levels (basic management units at the county level). TB designated hospitals at the county level provide TB diagnosis and treatment services. Diagnosed people are registered in web-based TB information management system (TBIMS). People on treatment make monthly visits to TB designated hospitals. Township clinics and village doctors (village level licensed general practitioners) assist in the continuation of treatment.

#### Study sites

China CDC developed an EMM scale-up plan for three provinces (involving 138 counties). Thirty counties were initially included in May and June 2018; other counties (n = 108) were involved by January 2019. In this study, we have reported the implementation experience from the first 30 counties which come from three provinces: nine from the Zhejiang province—east region, sixteen from Jilin province—middle region and five from Ningxia Autonomous Region—west region (**S1 Table**).

#### TB treatment

People with TB (not known to be rifampicin-resistant) receive daily fixed-dose combination treatment over six to eight months depending on whether they are newly diagnosed or previously treated (**S2 Table**). China’s NTP recommends two treatment adherence support systems: i) DOT (by township or village doctor or family member or volunteer) and ii) SAT with or without support by digital adherence technologies. If on SAT, the village doctors visit patients every ten days during the first two months of treatment followed by once a month to check on their health status and treatment adherence. Treatment outcomes are updated in the TBMIS at the county level.

#### Indigenously developed EMM

The Chinese NTP recommended an indigenously developed EMM [16]. The EMM was designed to monitor treatment adherence, in which could filled a one-month fixed-dose regimen. Also, the device alerted user through audible and visual alerts of daily dosing, monthly refill, and low battery.

**Fig 1:**
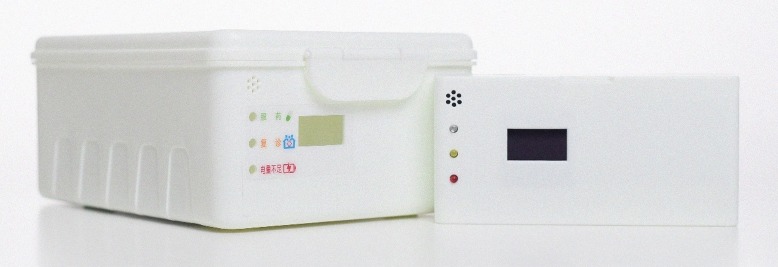
Picture of the electronic medication monitor device used for notified people with TB in the 30 selected counties of China. * *size: 16cm×12.5cm×7.3cm.

The device is relatively small (size: 16cm×12.5cm×7.3cm) and components included a plastic box and an electronic module. The electronic module records each time the patient opens the device, indicating the patient has taken his or her medication. This removable electronic module is reused for at least three treatment cycles (**Fig 1**). The battery works for at least two months, and the average cost per patient is approximately US$5.

A local producer was selected by tender. The detailed quality control protocol were developed and implemented during procurement and use. Before the devices were dispatched to each county, a qualified third-party agency tested the EMM; the tests included repeated pressing test, drop test, constant heat and humidity test, anti-vibration test, temperature cycle test, solar radiation test, and battery power test. During the use of the devices, a regular faulty device report system was established. However, no such report of faulty device was received during the study period.

#### EMM to support SAT

The standard operating procedure was developed and all the doctors from TB designated hospitals were trained. EMM supported SAT was offered to all the eligible people at the beginning of outpatient treatment. People having communication impairment (mental, visual, auditory, or speech) and requiring admission at notification were not eligible to use EMM.

Doctors programmed the EMM devices to provide a medication reminder at the same time (decided by the patient) every day. During the monthly patient visits, the adherence report was generated for the last month in the EMM operating system (offline software). If <20% of doses were missed, the patient was counselled on importance of treatment adherence. If 20–49% of doses were missed, the frequency of home visits by village doctors was increased to once every seven days for rest of the treatment. If there was continued instance of missing 20–49% of doses or a single instance of missing ≥50% of doses, the patient was shifted to DOT [16].If a patient missed the monthly visits or forgot to bring the EMM, the adherence status for the last month could not be assessed. In line with guidelines, doctors encouraged people to continue using EMM even after shifting to DOT.

The data from the EMM operating system were regularly uploaded to the online EMM information management system (EMM-IMS) by county-designated hospital staff. The progress report was developed and feedback provided on a monthly basis by the NTP.

### Study population

We included all notified people with TB in the 30 selected counties of China between July and December 2018. We excluded people known to be rifampicin-resistant or multidrug-resistant. We excluded people transferred into a county.

### Data variables and sources of data

We extracted data variables from TBIMS (notification date, diagnosis date, treatment initiation date, sex, age, occupation, migrant status, category of TB, classification of TB), EMM-IMS (date of starting and stopping of EMM, number of days the doses missed, number of days patient should take medicines from first to eight month) and paper-based patient records at county (eligible for EMM, reasons for non-eligibility, shifted to DOT anytime during treatment along with date of shifting).

### Data management and analysis

We digitized the data from the paper-based patient records (single-entered in Microsoft Excel (Microsoft, Redmond, WA, USA)) and merged it with TBIMS and EMM-IMS electronic databases using the TBIMS notification number. We used STATA (version 12.1, copyright 1985–2011 Stata Corp LP USA) for analysis.

We described the number (proportion) of eligible people. Among eligible, we derived EMM use within the first month of diagnosis based on dates of diagnosis and start of EMM use (considering some people need inpatient treatment but usually within one month). We calculated adjusted prevalence ratios and 95% confidence intervals (CI) using log-binomial regression to assess factors associated with not using EMM within the first month among all eligible people. We included all available variables in the regression model after ruling out multicollinearity (variance inflation factor ≥10). Among eligible and those who did not use EMM within the first month, we described the number and proportion that started using EMM later during treatment.

For every patient using EMM within the first month, we calculated month-wise missed doses as a proportion of the expected doses. We classified this to <20%, 20–49% and ≥50%. The follow-up period ended with the date of shift to DOT for those shifted to DOT and with the date of stopping EMM for those who were not shifted to DOT.

We described the number (proportion) eligible for shifting to DOT and actually shifted to DOT stratified by those who started using EMM within first month and after first month.

### Ethics

The ethics committee of the China CDC (no 201909 dated 18 April 2019) and the Ethics Advisory Group of the International Union Against Tuberculosis and Lung Disease (The Union), Paris, France (EAG no 15/19 dated 01 April 2019) approved the study. As the study involved use of secondary programme data, we sought waiver for informed consent and this was approved by the ethics committee(s).

## Results

### Patient characteristics

We identified 2294 notified people with TB, among them, the mean (standard deviation) age was 50.7 (18.4) years, 1565 (68.2%) were males and 1055 (46.0%) were farmers or herdsmen. Of the total, 130 (5.7%) were previously treated for TB and 1248 (54.4%) were bacteriologically confirmed. (**Table 1)**

**Table 1:** Socio-demographic, clinical and treatment accessibility related characteristics of notified people with TB in the 30 selected counties of China between July and December, 2018.

Characteristics		N	(%)
Total		2294	(100.0)
**Socio-demographic**			
Age in years			
	<15	9	(0.4)
	15–44	758	(33.0)
	45–64	883	(36.3)
	≥65	694	(30.3)
Sex			
	Male	1565	(68.2)
	Female	729	(31.8)
Occupation			
	Farmers and herdsmen	1055	(46.0)
	Semi-skilled employee	190	(8.3)
	Salary employee	238	(10.4)
	Unemployed	597	(26.0)
	Studying	115	(5.0)
	Others	99	(4.3)
Migrant*			
	No	2182	(95.1)
	Yes	112	(4.9)
**Clinical**			
Classification			
	Bacteriologically confirmed PTB	1248	(54.4)
	Clinically diagnosed PTB	840	(36.6)
	Pleurisy	206	(9.0)
Category			
	New	2164	(94.3)
	Previously treated	130	(5.7)
**Treatment accessibility**			
Treatment initiation interval (days)^*#*^			
	Zero	2191	(95.5)
	≥ One	103	(4.5)

Column percentages

TB–tuberculosis; PTB–pulmonary TB; HIV–human immunodeficiency virus

* Migrant defined as patients staying in the same prefecture for less than six months

^#^ Time interval from diagnosis to treatment, the median interval was zero days and used as cut-off to categorize

### EMM eligibility and uptake

**Fig 2:**
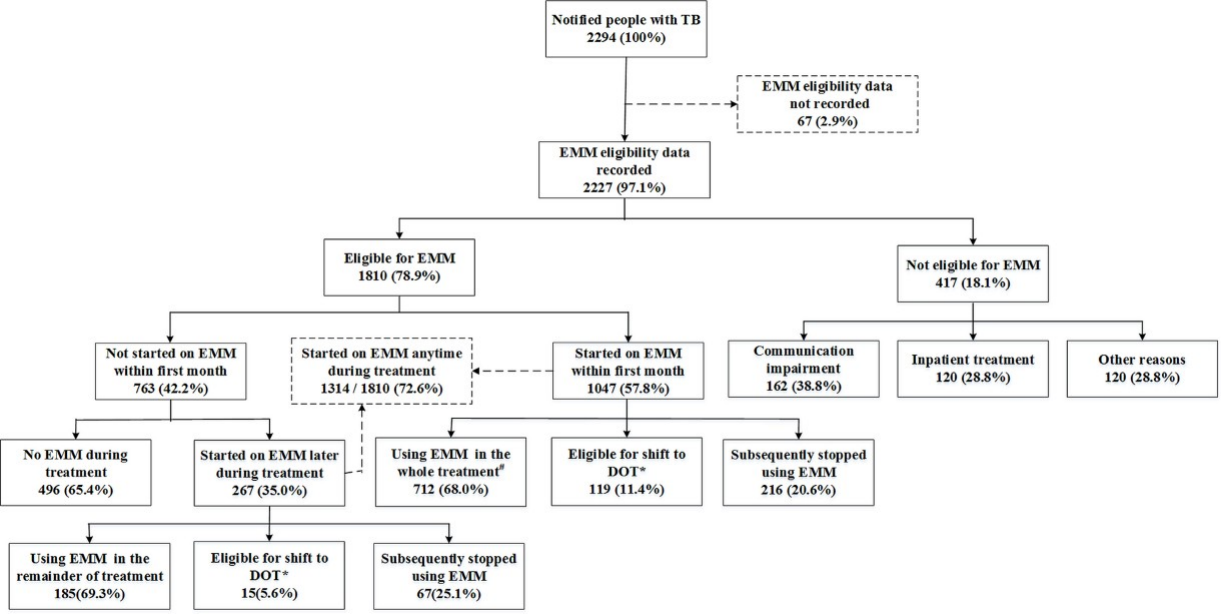
The flow chart depicting EMM eligibility, uptake and adherence among notified people with TB in the 30 selected counties of China between July and December, 2018. TB–tuberculosis; EMM–electronic medical monitor; DOT- directly observed treatment # Using EMM more than 6 month for new patients and 8 month for previously treated patients, besides there was no evidence of non-adherence * Continued instance of missing 20–49% of doses or a single instance of missing ≥50% of doses in a month.

Data on EMM eligibility and uptake is depicted in **Fig 2**. Of 2294 people, 2227 (97.1%) had EMM eligibility related data. Of 2227 people, 417 (18.7%) were not eligible for EMM, among them, 162 (38.8%) had communication impairment, 135 (32.4%) needed inpatient treatment, and for 120 (28.8%) reasons were unknown.

Of the 1810 EMM eligible people, a total of 1314 (72.6%) ever used EMM anytime during the treatment, 1047 (57.8%) started using EMM within the first month and 833 (46%) started using EMM at the diagnosis day.

### Factors associated with not using EMM within the first month

On adjusted analysis, children (<15 years), elderly (≥65 years), semi-skilled or unemployed people, people with tuberculosis pleurisy and previous tuberculosis treatment were less likely to use EMM within the first month **(Table 2)**.

**Table 2:** Factors associated with not using EMM^ within first month of diagnosis among eligible notified people with TB in the 30 selected counties of China between July and December, 2018^^.

Factors		Total	Not using EMM^		PR	(95% CI)	aPR^@^	(95% CI)
			n	(%)				
Total		1810	763	(42.2)	-	-	-	-
Age in years								
	<15	8	5	(62.5)	1.6	(0.90–2.69)	1.9	(1.01–3.39)*
	15–44	629	252	(40.1)	ref		ref	
	45–64	667	274	(41.1)	1.0	(0.90–1.17)	1.1	(0.91–1.19)
	> = 65	506	232	(45.9)	1.1	(1.00–1.31)*	1.3	(1.13–1.54)*
Sex								
	Male	1221	505	(41.4)	ref		ref	
	Female	589	258	(43.8)	1.1	(0.95–1.19)	1.1	(0.95–1.19)
Occupation								
	Farmers and herdsmen	816	309	(37.9)	ref		ref	
	Semi-skilled employee	168	71	(42.3)	1.1	(0.92–1.36)	1.2	(1.01–1.52)*
	Salary employee	194	93	(47.9)	1.3	(1.07–1.50)*	1.1	(0.97–1.37)
	Unemployed	450	217	(48.2)	1.3	(1.12–1.45)*	1.2	(1.02–1.35)*
	Studying	100	38	(38.0)	1.0	(0.77–1.31)	0.9	(0.70–1.27)
	Others	82	35	(42.7)	1.1	(0.86–1.47)	1.2	(0.91–1.57)
Migrant**								
	No	1733	736	(42.5)	1.2	(0.89–1.65)	1.2	(0.86–1.61)
	Yes	77	27	(35.1)	ref		ref	
Classification								
	Bacteriologically confirmed PTB	980	396	(40.4)	ref		ref	
	Clinically diagnosed PTB	672	278	(41.4)	1.0	(0.91–1.15)	1.1	(0.97–1.23)
	Pleurisy	158	89	(56.3)	1.4	(1.19–1.63)*	1.6	(1.34–1.83)*
Category								
	New	1716	708	(41.3)	ref		ref	
	Previously treated	94	55	(58.5)	1.4	(1.19–1.70)*	1.6	(1.32–1.88)*
Treatment initiation interval (days)^*#*^								
	Zero	1731	729	(42.1)	ref		ref	
	≥ One	79	34	(43.0)	1.0	(0.79–1.32)	1.2	(0.89–1.52)

Row percentages

TB–tuberculosis; PTB–pulmonary TB;EMM- Electronic Medication Monitor; PR–prevalence ratio; aPR–adjusted prevalence ratio; CI–confidence interval

^ Includes those who used EMM later during treatment or never used EMM during treatment

**^^** Of 2294 notified people with TB, 2227 has EMM eligibility data recorded. Of 2227, 1810 were eligible to use EMM at notification. Of 1810, 1047 used EMM within first month of diagnosis.

^**@**^ Adjusted prevalence ratios (95% CI) calculated using log binomial regression. We included all the variables including county in the multivariable analysis (county not shown in the table)

* Statistically significant

** Migrant defined as patients staying in the same prefecture for less than six months

# Time interval from diagnosis to treatment, the median interval was zero days and used as cut-off to categorize

### Treatment adherence and shift to DOT

Of the 1047 people who used EMM within the first month, 712 (68%) used EMM throughout treatment, 119 (11.4%) were eligible for the shift to DOT and 216 (20.6%) subsequently stopped using EMM. We observed a total of 6381 person-months of follow up (median months with EMM data available per person was six (inter-quartile range: 3–7)), and of them, there were 4607 (72.2%) instances of <20% missed doses, 173 (2.7%) instances of 20–49% missed doses, 75 (1.2%) instances ≥50% missed doses and 1526 (25.1%) instances where the adherence was not known (no EMM data) **(Table 3).**

**Table 3:** Treatment adherence measured as monthly proportion of missed doses among notified people with TB using EMM within first month of diagnosis in the 30 selected counties of China between July and December, 2018 [N = 1047].

Treatment in month	People at start (N)	Proportion of missed doses							
		Not known*		0–19%		20–49%		≥50%	
		n	(%)	n	(%)	n	(%)	n	(%)
1 month	1047	168	(16.0)	816	(77.9)	49	(4.7)	14	(1.3)
2 month	1004	163	(16.2)	786	(78.3)	45	(4.5)	10	(1.0)
3 month	921	167	(18.1)	716	(77.7)	25	(2.7)	13	(1.4)
4 month	884	182	(20.6)	672	(76.0)	21	(2.4)	9	(1.0)
5 month	843	203	(24.1)	619	(73.4)	13	(1.5)	8	(0.9)
6 month	802	212	(26.4)	575	(71.7)	10	(1.2)	5	(0.6)
7 month	576	230	(39.9)	322	(55.9)	9	(1.6)	15	(2.6)
8 month	304	201	(66.1)	101	(33.2)	1	(0.3)	1	(0.3)
Overall during treatment (patient-months)^#^	6381	1526	(23.9)	4607	(72.2)	173	(2.7)	75	(1.2)

Row percentages

TB–tuberculosis; EMM- Electronic Medication Monitor

* EMM could not offer anti-TB treatment adherence data, the reason involved incorrect usage by doctor/patients; patient did not bring EMM; patient did not make the monthly visit to TB designated hospital; faulty devices but not reported

**#** Cumulative patient-months

Of 267 started using EMM after the first month, 185 (69.3%) continued using EMM throughout treatment, 15 (5.6%) were eligible for the shift to DOT, and 67 (25.1%) subsequently stopped using EMM (**Fig 2**).

**Fig 3:**
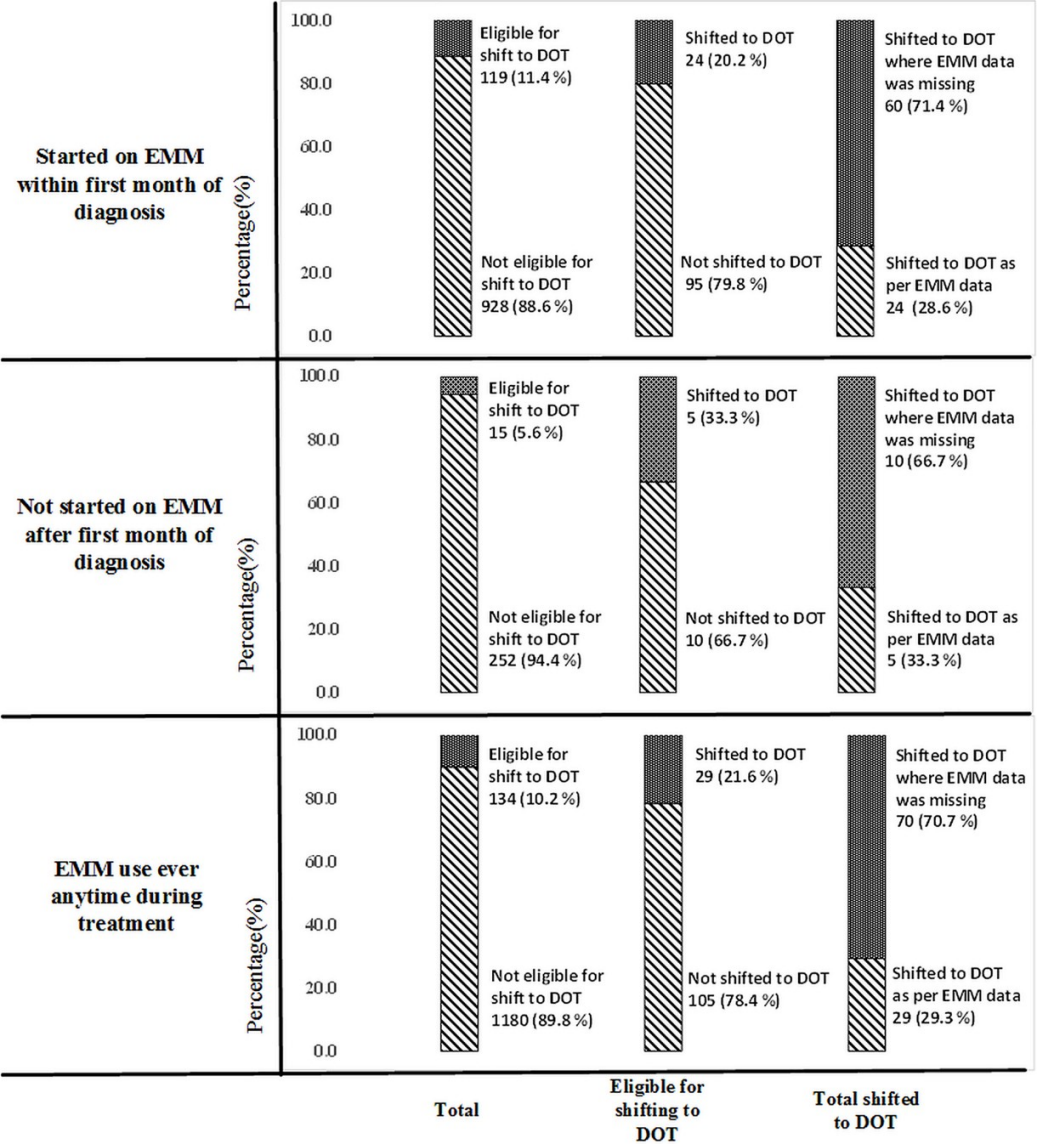
Shift to DOT among people with TB using EMM in the 30 selected counties of China between July and December, 2018*. TB–tuberculosis; EMM–electronic medical monitor; DOT- directly observed treatment * Of 2294 notified people with TB, 2227 has EMM eligibility data recorded. Of 2227, 1810 were eligible to use EMM at notification. Of 1810, 1047 used EMM within first month of diagnosis. Of 763 that did not use EMM within first month, 267 used EMM later during treatment.

Of 1314 using EMM (ever) anytime during the treatment, 134 (10.2%) were eligible for shift to DOT (based on EMM data) and of them, 29 (21.6%) were shifted. In addition, 70 people were shifted to DOT while EMM data was missing **(Fig 3)**.

## Discussion

### Key findings

Globally, this is the first study on large scale implementation experience of an EMM that does not provide real-time data. The findings will support and improve the further expansion of this locally developed, low-cost, quality-assured device. We found high patient eligibility and uptake. Specific groups that were less likely to be use EMM were identified. A low proportion of people was eligible for shift to DOT. Keeping in mind that instances of missing EMM data were common, the true proportion of eligibility for shift to DOT could be higher than reported. Action taken by the doctors for people who were eligible for the shift to DOT was limited.

### Limitations

There were some limitations. First, we did not know the reasons for missing EMM data. Any of the four possibilities were possible i) using EMM but patient did not make monthly visits ii) using EMM and patient made the monthly visits but EMM data was missing (incorrect usage by doctor/patients, did not bring EMM, brought EMM but data not uploaded to EMM-MIS) iii) patient not using EMM and iv) faulty devices but not reported. Because of significant instances of missing EMM data, we did not analyse the factors associated with being eligible for shift to DOT and stopping the use of EMM. Second, reasons for non-eligibility (for one-third of patients) and stopping EMM were not routinely recorded. Finally, as this was an operational research using data routinely recorded in programme setting, we cannot rule out recording errors.

### Interpretation of key findings

Limitations notwithstanding, there were some key findings. First, we found high eligibility and uptake of EMM. The uptake was satisfactory, considering other management options available with the patient. In addition, among those who used EMM within one month, four in five people started using EMM from the first day of treatment. This also supports the acceptability reported in trial settings in China [8]. This is promising, as the risk of treatment interruption is highest during the intensive phase of treatment [17].

Second, EMMs were less likely to be used by specific groups at the start of treatment and we need to pay more attention to these people who may also be more likely to have poor adherence and treatment outcomes: children, elderly, semi-skilled and unemployed people, people with TB pleurisy and previous TB treatment [4,18–20]. Though the limited acceptability of the device due to its inconspicuous nature was concluded in another studies [7,21]; there is no evidence (from our study) with which to conclude that specific sub-groups were concerned about the inconspicuous nature of the device.

Third, a significant number of people stopped EMM midway. A systematic review found the effectiveness of interventions to improve adherence decreased with every month [22], suggesting that people with TB need more interventions and feedback to enhance treatment adherence in the middle and later stages of treatment [23].

Fourth, the treating doctors did not shift people to DOT in nearly four-fifth instances when objective evidence was available. At the national level, it was difficult to monitor this based on monthly EMM-IMS reports. Though the number eligible for the shift to DOT was available in EMM-IMS, the number actually shifted was not available (available only in patient’s paper-based records). EMM-IMS only provided data on the number who stopped EMM use. However, as the guidelines recommended the continuation of EMM even after shift to DOT, this further made it difficult to assess shift to DOT based on EMM stop date objectively. A study from China also found that doctors from county-designated hospitals largely ignored the adherence data when deciding whether to switch people to a more intensive case management approach because of insufficient financial incentives [8]. In India, the healthcare providers also took no action even though they received alert messages from 99DOTS portal (99DOTS, which required patients to give a missed phone call after consuming drugs), because they believed it was due to incorrect usage by patients (inability to give a missed call) [24].

Finally, missing EMM data was common. Though there could be many reasons for this, there was no specific recommendation within guidelines regarding what needed to be done once this happened. We did find 70 instances where people were shifted to DOT in the absence of EMM data for the previous month. It is possible that some doctors were treating absence of the EMM data as a proxy for poor adherence. If that was the case and systematically implemented, the number of shifts to DOT should have been much more than reported.

### Implications for policy and practice

In programme settings in China, SAT with EMM has provided a useful alternative patient management option. The uptake of EMM appears to be satisfactory. For this to transform to better treatment outcomes, we recommend the following.

First and foremost, treating doctors should be encouraged and sensitized (by on-site and routine training) to take action based on EMM data and shift people to DOT as and when indicated. We speculate that action is similarly not being taken for <20% (patient counseling) and 20–49% missed doses (increasing frequency of village doctor home visits) and this has to be looked into. The integration of adherence data into counselling is paramount [25]. Second, EMM guidelines should be clear about what the treating doctor should do in case of missing EMM data. We recommend that these should be assumed as ≥50% of missed doses and shifted to DOT (assuming a worst-case scenario). Third, the date of the shift to DOT and reasons for stopping EMM should be included in the EMM-IMS. Finally, at the prefecture, province and national level, once the above recommendations are implemented, feedback should be provided in the progress report regarding the action taken among people eligible for shift to DOT.

Systematic qualitative enquiry is needed in order to understand how both providers and patients use the EMM, and if it is not being used as intended, why not? This will allow the device to gather more accurate adherence data. Additional research should also pay attention to factors associated with ceasing use of the EMM and eligibility for shift to DOT. Future research can then be conducted to assess the effect on TB treatment outcomes.

## Conclusion

Since the universal implementation of DOT in a country like China is impossible, China’s NTP intends to adopt EMM to support the SAT as a part of differential TB patient management approaches. In this study, we reported the implementation experience from the first 30 counties where SAT with EMM was suggested for all notified people with TB. The findings of this study have a number of important implications for improving this new patient management mechanism. Treatment adherence data should be actively used to identify and shift those who are eligible for DOT. This is essential to reap the benefits of this indigenously developed technology.

## Supporting information

S1 Table Summary of the study sites (30 selected counties that started implementing EMM by June 2018) to assess the uptake of EMM, adherence to treatment using EMM and the effect on TB treatment outcomes. (DOCX)

S2 Table Treatment regimens for people with TB used in China (2018–19). H: isoniazid; R: rifampicin; Z: pyrazinamide; E: ethambutol; S: streptomycin
(DOCX)

S3 Table Data set including the codebook. (XLSX)
