# Supplementary material for: Electronic medication monitor for people with tuberculosis: Implementation experience from thirty counties in China
Source: PLoS One. 2020 Apr 29;15(4):e0232337. doi: 10.1371/journal.pone.0232337 (PMC7190174; doi:10.1371/journal.pone.0232337)
Supplement: S1 Table — (DOCX) [file pone.0232337.s001.docx]

**S1 Annex. Summary of the study sites (30 selected counties that started implementing EMM by June 2018) to assess the uptake of EMM, adherence to treatment using EMM and the effect on TB treatment outcomes**

| **Region** | **Province** | **Number of counties** | **Name of the counties** |
| --- | --- | --- | --- |
| Eastern | Zhejiang | 9 | Qujiang, Changshan, Longyou, Kaihua, Jiangshan, Kecheng, Haiyan, Pinghu, Tongxiang |
| Middle | Jilin | 16 | Tonghua, Huinan, Dongchang, Jian, Liuhe, Meihekou, Daan, Fusong, Linjiang, Xian ,Tiedong, Huichun, Dunhua, Yanji, Lvyuan, Erdao |
| West | Ningxia | 5 | Dawukou, Pingluo, Lingwu, Yongning, Yanchi |
