# Supplementary material for: Electronic medication monitor for people with tuberculosis: Implementation experience from thirty counties in China
Source: PLoS One. 2020 Apr 29;15(4):e0232337. doi: 10.1371/journal.pone.0232337 (PMC7190174; doi:10.1371/journal.pone.0232337)
Supplement: S2 Table — H: isoniazid; R: rifampicin; Z: pyrazinamide; E: ethambutol; S: streptomycin (DOCX) [file pone.0232337.s002.docx]

**S2 Annex. Treatment regimens for TB patients used in China (2018-19)**

| **Type** | **Regimen** |
| --- | --- |
| New TB | 2HRZE/4HR |
| Previously treated TB | 2HRZES/6HRE or 3HRZE/6HRE |
| Pleurisy | 2HRZE/7HRE or 2HRZE/10HRE |

H: isoniazid; R: rifampicin; Z: pyrazinamide; E: ethambutol; S: streptomycin
